# Supplementary material for: The History of the Brazilian Sardine (Sardinella brasiliensis) Between Two Fishery Collapses: An Ecosystem Modeling Approach to Study Its Life Cycle
Source: Biology (Basel). 2024 Dec 27;14(1):13. doi: 10.3390/biology14010013 (PMC11763292; doi:10.3390/biology14010013)
Supplement: Supplementary file 1 [file biology-14-00013-s001.zip › biology-3357322-supplementary.pdf]

Table S1. Diet composition matrix expressed in percent volume of prey groups of the main species landed in the *Sardinella brasiliensis* purse-seine fishery in the southeast and south Brazil. SL, Atlantic thread herring; BL, Skipjack tuna; DO, Dolphin fish; ES, Largehead hairtail; GU, Castin leatherjacket; OL, Yellowfin amberjack; XI, Rough scad; OC, Atlantic bigeye; TA, Lebranche mullet; GA, Atlantic moonfish; XA, Crevalle jack; PA, Atlantic bumper; CA, Atlantic chub mackerel; SC, False herring; CO, Whitemouth croaker; SV, Brazilian sardine, and the prey contained in their diet, PEP, pelagic fish; CEF, cephalopods; LUL, squids; PAB, benthic feeding fish; OBD, detritivores benthic organisms; OBC, carnivorous benthic organisms; PAP, pelagic feeding fish; CAR, crabs; EQU, echinoderms; INB, benthic invertebrates; CAM, shrimps; MOL, mollusks; ZOO, zooplankton; CNI, cnidarians; POL, polychaeta; BAP, bacterioplankton; ALG, algae; FIT, phytoplankton; DET, detritus.

Prey/Predator		1	2	3	4	5	6	7	8	9	10	11	12	13	14	15	16	17	18	19	20	21	22	23	24	25	26	27	28	29	30	31	32
1	SL	-	-	-	-	-	-	-	-	-	-	-	-	-	-	-	-	-	-	-	-	-	-	-	-	-	-	-	-	-	-	-	-
2	BL	-	-	0.0780	-	-	-	-	-	-	-	-	-	-	-	-	-	-	-	-	-	-	-	-	-	-	-	-	-	-	-	-	-
3	DO	-	-	0.0157	-	-	-	-	-	-	-	-	-	-	-	-	-	-	-	-	-	-	-	-	-	-	-	-	-	-	-	-	-
4	ES	-	-	0.1115	-	-	-	-	-	-	-	-	-	-	-	-	-	-	-	-	-	-	-	-	-	-	-	-	-	-	-	-	-
5	GU	-	-	-	-	-	-	-	-	-	-	-	-	-	-	-	-	-	-	-	-	-	-	-	-	-	-	-	-	-	-	-	-
6	OL	-	-	0.0019	-	-	-	-	-	-	-	-	-	-	-	-	-	-	-	-	-	-	-	-	-	-	-	-	-	-	-	-	-
7	XI	-	-	0.1341	-	-	0.3111	-	-	-	-	-	-	-	-	-	0.0013	-	-	-	-	-	-	-	-	-	-	-	-	-	-	-	-
8	OC	-	-	0.0031	-	-	-	-	-	-	-	-	-	-	-	-	-	-	-	-	-	-	-	-	-	-	-	-	-	-	-	-	-
9	PEP	-	0.5300	0.3394	-	-	0.0361	-	-	-	-	-	-	-	-	0.3500	0.9446	-	-	-	-	-	-	0.9300	-	-	-	-	-	-	-	-	-
10	TA	-	-	-	-	-	-	-	-	-	-	-	-	-	-	-	-	-	-	-	-	-	-	-	-	-	-	-	-	-	-	-	-
11	GA	-	-	-	-	-	-	-	-	-	-	-	-	-	-	-	0.0001	-	-	-	-	-	-	-	-	-	-	-	-	-	-	-	-
12	XA	-	-	0.0031	-	-	-	-	-	-	-	-	-	-	-	-	-	-	-	-	-	-	-	-	-	-	-	-	-	-	-	-	-
13	PA	-	-	-	-	-	-	-	-	-	-	-	-	-	-	-	-	-	-	-	-	-	-	-	-	-	-	-	-	-	-	-	-
14	CA	-	-	-	-	-	0.0890	-	-	-	-	-	-	-	-	-	-	-	-	-	-	-	-	-	-	-	-	-	-	-	-	-	-
15	CEF	-	0.1524	0.0415	-	-	-	-	-	-	-	-	-	-	-	0.1000	0.0106	0.0050	-	-	-	-	-	-	0.1000	-	-	-	-	-	-	-	-
16	LUL	-	0.0558	0.0019	0.1350	0.0010	0.1472	-	-	-	-	-	-	-	-	-	0.0000	-	-	-	-	-	-	-	-	-	-	-	-	-	-	-	-
17	PAB	-	-	0.1722	-	-	0.0098	-	-	-	-	-	-	-	-	-	0.0000	0.0540	-	0.0244	-	-	-	-	0.0500	-	-	-	-	-	-	-	-
18	SC	-	-	-	-	-	-	-	-	-	-	-	-	-	-	-	-	-	-	-	-	-	-	-	-	-	-	-	-	-	-	-	-
19	CO	-	-	-	-	-	-	-	-	-	-	-	-	-	-	-	-	-	-	-	-	-	-	-	-	-	-	-	-	-	-	-	-
20	SV	-	0.0558	0.0516	-	-	-	-	-	-	-	-	-	-	-	-	0.0003	-	-	-	-	-	-	-	-	-	-	-	-	-	-	-	-
21	OBD	-	-	-	-	-	-	-	-	-	-	0.0001	-	-	-	0.1500	-	0.1500	-	-	-	0.0300	0.1200	0.0200	0.1000	0.0500	0.4490	0.2000	-	-	0.1500	-	-
22	OBC	-	-	-	-	-	-	-	-	-	-	-	-	0.0015	-	-	-	-	-	-	-	-	0.0400	-	0.0500	0.0100	0.1973	-	-	-	-	-	-
23	PAP	-	0.0558	0.0019	0.7630	0.0240	0.4069	0.0005	-	-	-	0.0220	-	0.0226	0.1720	0.0300	0.0009	-	0.0132	-	-	-	-	-	-	-	-	-	-	-	-	-	-
24	CAR	-	-	-	-	-	-	0.0098	-	-	-	-	-	0.0632	-	0.1500	-	0.0600	-	-	-	-	-	0.0030	0.0500	-	-	-	-	-	-	-	-
25	EQU	-	-	-	-	-	-	-	-	-	-	-	-	-	-	-	-	0.1400	-	-	-	-	0.0850	-	0.1000	-	-	0.0300	-	-	0.0300	-	-
26	INB	-	0.0687	-	-	-	-	-	0.3014	-	-	-	-	-	-	-	0.0404	-	-	0.1377	-	-	-	-	-	-	-	-	-	-	-	-	-
27	CAM	-	-	-	0.1020	0.8410	-	-	-	-	-	0.8740	1.0000	0.3711	-	0.1200	-	0.0900	-	-	-	-	-	0.0470	0.0500	-	0.3429	-	-	-	0.0200	-	-
28	MOL	-	0.0558	0.0006	-	-	-	0.0028	-	-	-	-	-	-	0.0260	-	-	-	-	0.7960	-	-	0.0500	-	0.0700	-	-	-	-	-	0.0050	-	-
29	ZOO	0.6600	0.0258	0.0393	-	0.1340	-	0.9870	0.2603	0.9000	-	0.1039	-	0.3345	0.7780	0.1000	-	-	0.6981	0.0000	0.7027	0.1000	-	-	-	0.1200	0.0107	0.2000	0.3000	0.0500	0.3400	-	-
30	CNI	-	-	0.0006	-	-	-	-	-	-	-	-	-	-	-	-	-	0.0010	-	0.0198	-	-	0.0050	-	-	-	-	-	-	-	-	-	-
31	POL	-	-	-	-	-	-	-	-	-	-	-	-	-	0.0240	-	0.0018	0.5000	-	0.0221	-	-	0.1500	-	0.0300	0.0600	-	0.1000	-	-	0.0050	-	-
32	BAP	-	-	-	-	-	-	-	-	-	-	-	-	-	-	-	-	-	-	-	-	0.0500	-	-	-	-	-	-	-	0.0500	-	-	-
33	ALG	-	-	0.0037	-	-	-	-	-	-	-	-	-	-	-	-	-	-	-	-	-	-	-	-	-	-	-	-	-	-	-	-	-
34	FIT	-	-	-	-	-	-	-	-	0.1000	-	-	-	-	-	-	-	-	0.2671	-	0.2973	0.0500	-	-	-	0.0600	-	-	0.2000	0.9000	-	-	-
35	DET	0.3400	-	-	-	-	-	-	0.4384	-	1.0000	-	-	0.2071	-	-	-	-	0.0215	-	-	0.7700	0.5500	-	0.4000	0.7000	-	0.4700	0.5000	-	0.4500	1.0000	1.0000
